# Redifferentiation of expanded human islet β cells by inhibition of ARX

**DOI:** 10.1038/srep20698

**Published:** 2016-02-09

**Authors:** Orr Friedman-Mazursky, Ran Elkon, Shimon Efrat

**Affiliations:** 1 Department of Human Molecular Genetics and Biochemistry, Sackler School of Medicine, Tel Aviv University, Tel Aviv, Israel

## Abstract

*Ex-vivo* expansion of adult human islet β cells has been evaluated for generation of abundant insulin-producing cells for transplantation; however, lineage-tracing has demonstrated that this process results in β-cell dedifferentiation. Redifferentiation of β-cell-derived (BCD) cells can be achieved using a combination of soluble factors termed Redifferentiation Cocktail (RC); however, this treatment leads to redifferentiation of only a fraction of BCD cells. This study aimed at improving redifferentiation efficiency by affecting the balance of islet progenitor-cell transcription factors activated by RC treatment. Specifically, RC treatment induces the transcription factors PAX4 and ARX, which play key roles in directing pancreas endocrine progenitor cells into the β/δ or α/PP developmental pathways, respectively. Misactivation of ARX in RC-treated BCD cells may inhibit their redifferentiation into β cells. Blocking ARX expression by shRNA elevated insulin mRNA levels 12.8-fold, and more than doubled the number of insulin-positive BCD cells. ARX inhibition in expanded α-cell-derived cells treated with RC did not cause their transdifferentiation into insulin-producing cells. The combination of RC and *ARX* shRNA treatment may facilitate the generation of abundant insulin-producing cells for transplantation into patients with type 1 diabetes.

Type 1 Diabetes Mellitus (T1DM) is caused by an autoimmune destruction of insulin-producing β cells, resulting in chronic hyperglycemia. The current treatment involves monitoring blood glucose levels and administration of insulin. However, due to the difficulties in maintaining appropriate glycemic levels, T1DM patients exhibit an increased risk of vascular complications^1^. Current research focuses on β-cell replacement as a therapy for T1DM. The major obstacle to this approach is the severe shortage of human organ donors. *In-vitro* expansion of human islet β cells represents an attractive strategy for generation of an abundant source of cells for β-cell replacement; however, massive islet cell proliferation is associated with a rapid loss of β-cell phenotype^2^,^3^. Using a genetic lineage-tracing approach based on lentivirus vectors we provided evidence for massive proliferation and dedifferentiation of human β-cell-derived (BCD) cells^4^, which is associated with a process resembling epithelial-mesenchymal transition (EMT)^5^. BCD cells, which constitute ~40% of islet cell cultures^4^, maintain open chromatin structure at β-cell genes^6^ and can be redifferentiated in response to a combination of soluble factors termed Redifferentiation Cocktail (RC)^7^. The redifferentiated cells re-express β-cell genes, process and store insulin in typical secretory vesicles, and release it in response to glucose. However, RC treatment leads to redifferentiation of only a fraction of BCD cells, raising the need for further improvements of redifferentiation methods.

Redifferentiation involves activation of transcription factors characteristic of islet progenitor cells, including SOX9, FOXA2, PDX1, NGN3, PAX4 and ARX^7^. Mouse gene knockout models helped elucidate the roles played by these factors during pancreatic development^8^. SOX9^9^, FOXA2^10^, and PDX1^11^ are activated in pancreatic progenitor cells and required for their early differentiation. Subsequently, the transcription factor NGN3 specifies the endocrine cell lineage^12^,^13^. Differentiation towards mature endocrine cells is largely dependent on additional transcription factors, including PAX4 and ARX. PAX4 is essential for differentiation of the β- and δ-cell lineages, while ARX is involved in the specification of the α- and PP-cell fate^14^,^15^,^16^. These two factors repress each other’s expression, thereby mediating the specification toward the endocrine subtype destinies.

In adult mice, ectopic expression of ARX in β cells lead to loss of β-cell phenotype and conversion into glucagon- or PP-producing cells^17^. Conversely, ectopic expression of PAX4 forced embryonic and adult α cells to adopt a β-cell phenotype^18^,^19^. Selective inhibition of *Arx* in α cells was sufficient for promoting the conversion of adult α cells into β-like cells in mice^20^. Analysis of *Arx* and *Pax4* conditional double-mutants provided evidence that PAX4 was dispensable for the α-to-β-cell conversion, indicating that ARX downregulation was the main trigger of this process^20^.

Bramswig *et al*. showed that genes active in β cells and repressed in α cells, such as *Pdx1* and *Ins*, could be activated by an inhibitor of histone methyltransferase^21^, thereby emphasizing the role of epigenetic modifications in α-to-β-cell transdifferentiation. Piran *et al*. observed transdifferentiation of α–to–β–to–δ cells in mice induced by acinar and β cells ablation^22^. Fomina-Yadlin *et al*. demonstrated α-to-β reprogramming *in vitro* in a murine α-cell line that was driven towards insulin-producing cells by a small-molecule inducer of insulin expression^23^. Another study by Yang *et al*. used ectopic PDX1 expression in murine embryonic and mature α cells to reprogram them into β cells^24^. Taken together these findings highlight the plasticity of α cells and offer hope for development of novel therapeutic paths for replenishing β cells in diabetes. However, a similar plasticity has not been demonstrated yet in human islet α cells.

In this study, we hypothesized that ARX misactivation in RC-treated BCD cells may limit their redifferentiation into insulin-producing cells. Our findings demonstrate that inhibition of ARX expression by shRNA leads to significantly enhanced BCD cell redifferentiation, as manifested by an increase in expression of insulin and the number of insulin-positive cells. In contrast to the effect seen in BCD cells, our data suggests that ARX inhibition in expanded α-cell-derived cells treated with RC does not lead to their transdifferentiation into β-like cells.

## Results

### Changes in *ARX* and *PAX4* expression in expanded adult human islet cells

**Figure 1:**
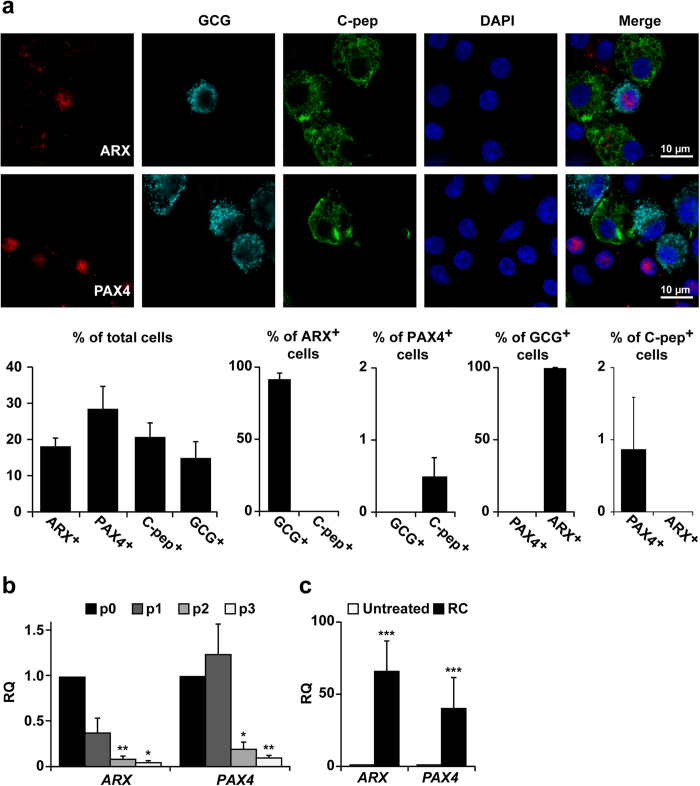
Changes in *ARX* and *PAX4* expression in expanded adult human islet cells. **(a)** Immunofluorescence analysis of uncultured dissociated islet cells. Data are mean ± SE (n = 3 donors), based on counting >500 cells per donor. ARX is co-expressed with GCG but not C-peptide, while PAX4 is expressed in GCG^-^ and C-peptide^-^ cells. The percentages of C-pep^+^ and GCG^+^ cells are lower than those observed in pancreas sections, given isolated islet purity, and expression loss during shipment. (**b)** qPCR analysis of RNA extracted from isolated islets (p0) and expanded islet cells at the indicated passage number. Data are mean ± SE (n = 4 donors). *p ≤ 0.05, **p ≤ 0.01, compared with p0. (**c)** qPCR analysis of RNA extracted from cells at passages 4–6 treated with RC for 8 days. Data are mean ± SE (n = 7 donors). ***p ≤ 0.001, compared with untreated cells.

In adult human islet cells ARX expression is restricted to α cells and is absent from β cells, while PAX4 is expressed in non-β and non-α cells (Fig. 1a). *ARX* and *PAX4* transcripts were significantly downregulated during the first three weeks of islet cell expansion *in vitro* (Fig. 1b), and upregulated following treatment with RC (Fig. 1c). The RC treatment was developed to induce redifferentiation of expanded islet cells into insulin-producing cells. Since ARX expression has been associated with development of α cells, we hypothesized that its activation by RC treatment may interfere with restoration of the β-cell phenotype. To evaluate this possibility *ARX* shRNA was employed to block ARX expression.

### Effects of *ARX* inhibition in expanded islet cells

**Figure 2:**
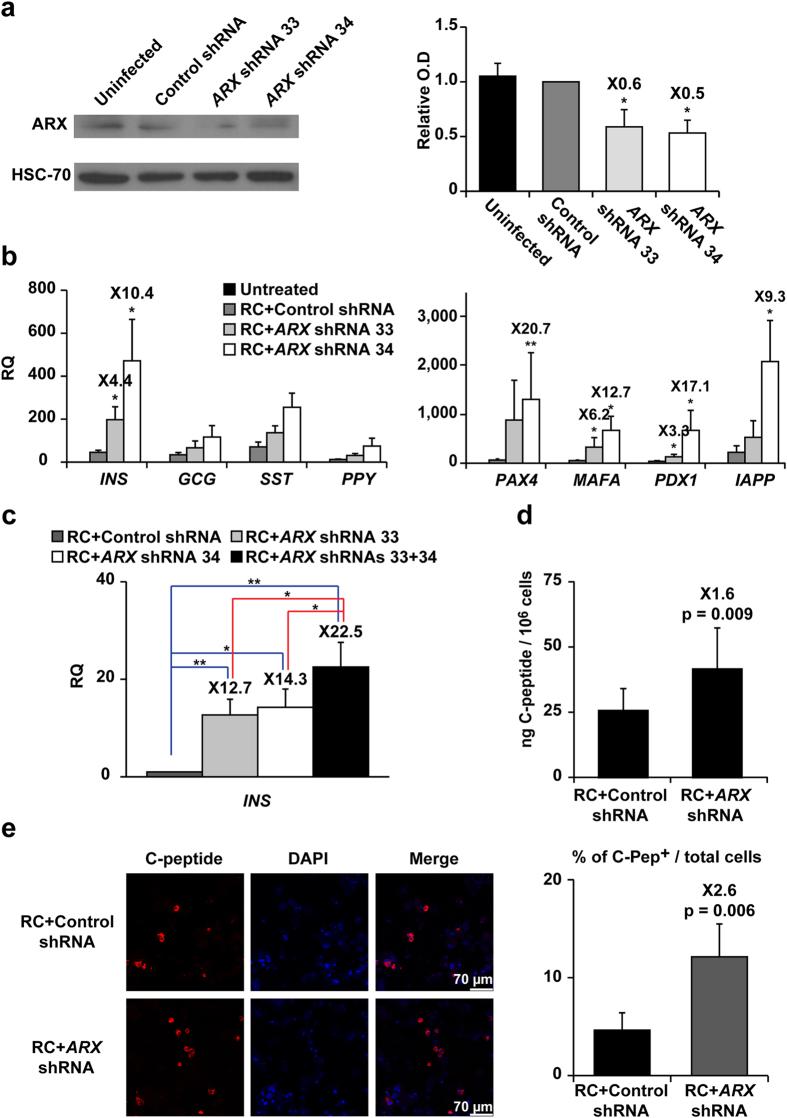
Effects of *ARX* inhibition in expanded islet cells. **(a)** Immunoblotting of ARX in expanded islet cells at passage 8, 6 days following infection with *ARX* or control shRNA viruses (cropped blot). HSC70 was used as a loading control. Data are mean ± SE (n = 3 donors). *p ≤ 0.05, compared with control shRNA. (**b)** qPCR analysis of RNA extracted from expanded islet cells infected at passages 5–6 with *ARX* or control shRNA viruses and treated with RC for 8 days. Data are mean ± SE (n = 3 donors). *p ≤ 0.05, **p ≤ 0.01, relative to RC+control shRNA. Values on top of bars indicate fold change relative to RC+control shRNA. (**c)** qPCR analysis of insulin transcripts in expanded islet cells infected at passages 5–7 with *ARX* or control shRNA viruses and treated with RC for 4 days. Data are mean ± SE (n = 3 donors). *p ≤ 0.05, **p ≤ 0.01. (**d)** C-peptide content of expanded islet cells infected at passages 3–4 with *ARX* or control shRNA viruses and treated with RC for 4 days. Data are mean ± SE (n = 4 donors). Value on top of bar indicates fold change relative to RC+control shRNA. (**e)** Immunofluorescence analysis of C-peptide in expanded islet cells infected at passages 3–4 with *ARX* or control shRNA viruses and treated with RC for 4 days. Data are mean ± SE (n = 5 donors), based on counting >1000 cells per donor. Value on top of bar indicates fold change relative to RC+control shRNA.

Two *ARX* shRNAs were evaluated for their inhibitory activity in expanded islet cells. Although these cells contain low *ARX* transcript levels, compared with uncultured islets (Fig. 1b), these levels were sufficient for evaluating the shRNA efficiency. The shRNAs reduced ARX protein levels in expanded islet cells by 40–50%, compared with cells treated with control shRNA (Fig. 2a). Analysis of expanded islet cells infected with *ARX* shRNA virus and treated with RC revealed a significant 4–10-fold increase in *INS* transcript levels (Fig. 2b). In contrast, transcripts for the other islet hormones were not significantly changed. The increase in *INS* transcripts was accompanied by induction of transcripts encoding the *INS* gene transcription factors PAX4, PDX1 and MAFA, as well as *IAPP* transcripts (Fig. 2b). Expression of *INS* transcript levels was significantly increased in cells treated with the combination of *ARX* shRNAs, compared to each shRNA alone (Fig. 2c). Based on this result, the combination of shRNAs was used in further work. Analysis of extracts of shRNA-infected cells following RC treatment revealed a 1.6-fold increase in C-peptide content in *ARX* shRNA-treated cells, compared to cells treated with control shRNA (Fig. 2d). The increase in protein level was low, compared with the large increase in transcript levels (Fig. 2c). The number of C-peptide^+^ cells was 2.6-fold larger in cells treated with *ARX* shRNA than in cells treated with control shRNA (Fig. 2e). This finding suggests that the major effect of ARX inhibition is an increase in the number of C-peptide-expressing cells, rather than an increase of C-peptide expression per cell. Considering the fraction of C-peptide^+^ cells in the cell population (12%), and the C-peptide content of normal islet β cells (3500 ng/10^6^ islet cells, of which on average 40% are β cells^7^), the content achieved with the combined treatment of *ARX* shRNA and RC represents an estimated 4% of C-peptide content of normal islet β cells.

### Effects of *ARX* inhibition in BCD cells

**Figure 3:**
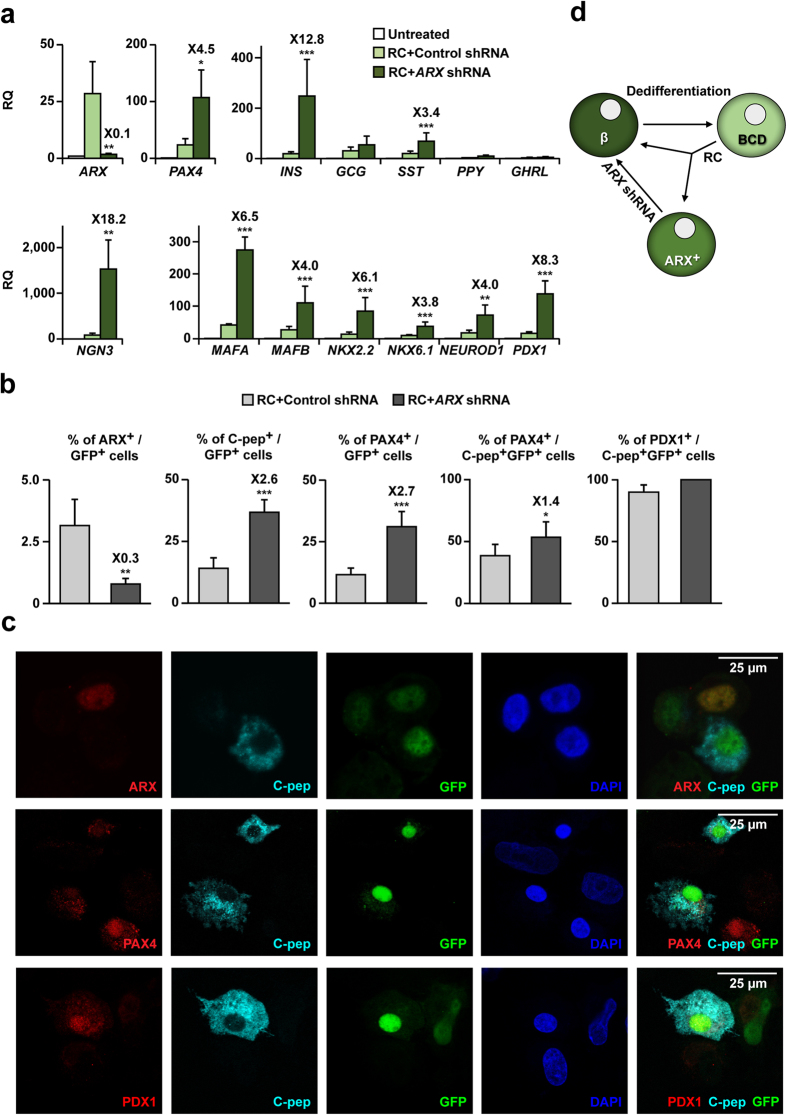
Effects of *ARX* inhibition in BCD cells. **(a)** qPCR analysis of RNA extracted from sorted GFP^+^ BCD cells infected at passages 6–8 with *ARX* or control shRNA viruses, and treated with RC for 4 days. Data are mean ± SE (n = 4–6 donors). (**b)** Immunofluorescence analyses of the indicated proteins in GFP^+^ BCD cells infected at passages 3–6 with *ARX* or control shRNA viruses, and treated with RC for 4 days. Values are mean ± SE (n = 3–5 donors), based on counting >1000 cells per donor. (**c)** Representative immunofluorescence images depicting ARX expression in GFP^+^, but not C-peptide^+^, cells; PAX4 expression in some C-peptide^+^ cells; and PDX1 expression in virtually all C-peptide^+^ cells. (**d**) Proposed model for redifferentiation of BCD cells by ARX inhibition. *p ≤ 0.05, **p ≤ 0.01, ***p ≤ 0.005, compared with RC+control shRNA.

The increase in the number of INS-expressing cells following inhibition of ARX expression may result from enhanced redifferentiation of BCD cells, transdifferentiation of non-BCD cells, or both. To test the possibility of enhanced redifferentiation of BCD cells, GFP^+^ BCD cells were sorted to an average purity of 88.4% (see Supplementary Fig. S1). Analysis of sorted BCD cells treated with control shRNA and RC showed an increase in *ARX* transcript levels, demonstrating that ARX expression was induced by RC in BCD cells (Fig. 3a). Treatment with *ARX* shRNA resulted in a 10-fold decrease in *ARX* transcript levels and a 12.8-fold increase in *INS* transcript levels, compared to cells treated with the control vector. *SST* transcript levels were also induced 3.4-fold, while transcripts for other islet hormones were not affected. *PAX4* transcript levels were upregulated 4.5-fold, in accordance with the mutual inhibition between *Pax4* and *Arx* in mouse endocrine pancreas development. Transcripts encoding the islet progenitor-cell transcription factor NGN3 were upregulated 18.2-fold. Transcripts encoding six other β-cell transcription factors were also significantly upregulated 3.8-8.3-fold (Fig. 3a). These findings demonstrate that *ARX* inhibition upregulates the expression of β-cell genes in BCD cells.

Analysis of expanded islet cells infected with *ARX* shRNA virus and treated with RC revealed a significant 70% reduction in ARX^+^ cells among GFP^+^ BCD cells, which was accompanied by a commensurate 2.6-fold increase in C-peptide^+^ cells, compared with cells treated with control shRNA (Fig. 3b), from 14.1 ± 4.2% to 36.8 ± 5.0% of GFP^+^ cells. ARX inhibition was also accompanied by a 2.7-fold increase in PAX4^+^ cells (Fig. 3b). Approximately half (53.6%) of C-peptide^+^ cells were also PAX4^+^ (Fig. 3b,c). Virtually all C-peptide^+^ cells were PDX1^+^ as well (Fig. 3b,c). These findings confirm the hypothesis that ARX is misactivated in some BCD cells treated with RC, and its downregulation promotes the differentiation of additional BCD cells. Accordingly, staining for C-peptide and ARX in BCD cells following treatment with RC was mutually exclusive (Fig. 3c), suggesting that ARX expression prevents the expression of INS, and its inhibition is needed for differentiation of these BCD cells into β-like cells (Fig. 3d).

**Figure 4:**
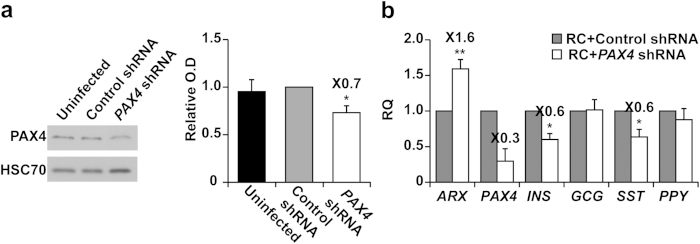
Effects of *PAX4* inhibition in expanded islet cells. **(a)** Immunoblotting of PAX4 in expanded islet cells infected at passages 5–6 with *PAX4* or control shRNA viruses (cropped blot). HSC70 was used as a loading control. Data are mean ± SE (n = 3 donors). *p ≤ 0.05, compared with control shRNA. (**b)** qPCR analysis of RNA extracted from expanded islet cells infected at passages 5–7 with *PAX4* or control shRNA viruses, and treated with RC for 4 days. Data are mean ± SE (n = 3–8 donors). *p ≤ 0.05, **p ≤ 0.005, relative to RC+control shRNA.

To confirm the role of PAX4 in the differentiation of β cells, as demonstrated by its elevation following ARX inhibition, the effect of PAX4 downregulation was evaluated. *PAX4* shRNA reduced PAX4 protein levels by 30%, compared with cells treated with control shRNA (Fig. 4a). Analysis of expanded islet cells infected with *PAX4* shRNA and treated with RC showed a significant 40% decrease in *INS* and *SST* transcript levels, while no change was observed in *GCG* and *PPY* transcript levels (Fig. 4b). Inhibition of PAX4 also resulted in a significant 1.6-fold increase in *ARX* transcript levels (Fig. 4b). These results demonstrate that inhibition of PAX4 has a negative effect on restoration of the β-cell phenotype in expanded human islet cells, in agreement with findings in rodent systems^14^.

**Figure 5:**
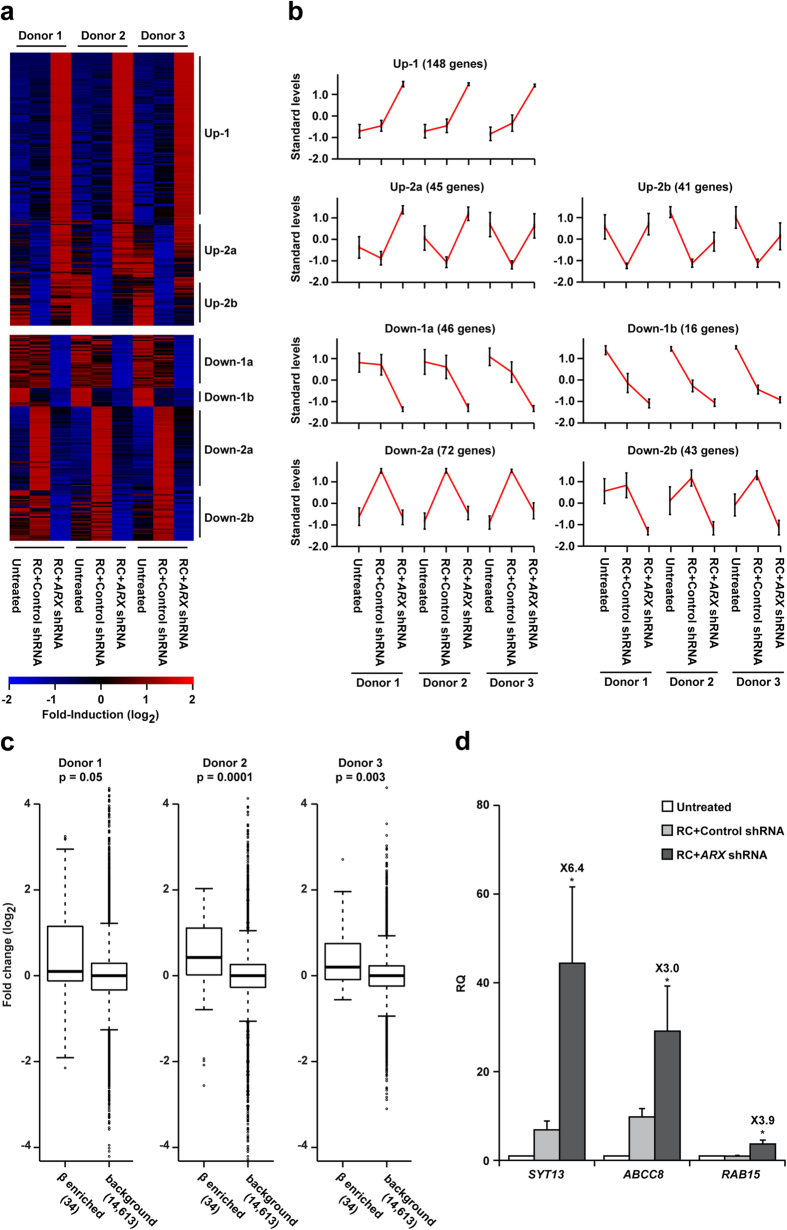
Analysis of global changes in gene expression in BCD cells treated with *ARX* shRNA and RC. RNA-seq analyses of RNA extracted from sorted GFP^+^ BCD cells infected at passages 5–6 with *ARX* or control shRNA viruses, and treated with RC for 4 days (n = 3 donors). (**a)** Heatmap and **b,** cluster analyses of genes upregulated or downregulated >1.5-fold in cells treated with RC+ *ARX* shRNA, compared with RC+control shRNA. (**c)** Comparison of the effect of *ARX* inhibition in a set of genes which characterize β cells, with that in all other genes in the dataset. p-values were calculated using Wilcoxon’s test. The boxes indicate the 1^st^ and 3^rd^ quartiles of the fold-change distribution; the band inside the box indicates the median. The whiskers extend to data points 1.5-times the interquartile range from the box. (**d)** qPCR validation of selected genes. Data are mean ± SE (n = 4–7 donors). *p ≤ 0.005. Values on top of bars indicate fold change relative to RC+control shRNA.

To obtain a global view of the effect of *ARX* inhibition on BCD cell redifferentiation, RNA-seq analysis was performed. The analysis identified 261 upregulated and 197 downregulated genes (>1.5-fold; n = 3 donors; FDR <0.1) in cells treated with RC+ *ARX* shRNA, compared with RC+ control shRNA. Cluster analysis identified two main patterns among upregulated genes: genes upregulated in response to RC and further upregulated upon *ARX* inhibition (Fig. 5a,b, cluster Up-1; see Supplementary Table S1), including the β-cell genes *INS*, *PDX1*, and *ABCC8*; and two clusters of genes inhibited by RC and partly or completely reactivated upon *ARX* inhibition (Fig. 5a,b, clusters Up-2a and Up-2b; see Supplementary Table S1), including *CXCL12* and *CTGF*, which encode proteins that promote β-cell survival and maturity^25^,^26^. Among downregulated genes, two clusters included genes suppressed in response to RC and further blocked upon *ARX* inhibition (Fig. 5a,b, clusters Down-1a and Down-1b; see Supplementary Table S2), including *SEPT5*, which encodes a protein that downregulates insulin secretion^27^, and two clusters included genes induced by RC and attenuated by *ARX* inhibition (Fig. 5a,b, clusters Down-2a and Down-2b; see Supplementary Table S2). Functional annotation revealed that cluster Up-1 was significantly enriched for genes encoding signaling and membrane proteins, while cluster Up-2 was enriched for genes encoding extracellular and secreted proteins (see Supplementary Fig. S2). Both clusters were enriched for genes encoding proteins with peptidase activity. No enriched categories were detected in gene clusters downregulated by *ARX* inhibition. In samples from all three donors *ARX* inhibition resulted in significant upregulation of a group of 34 genes defined as a molecular signature of β cells^28^ (Fig. 5c). To validate the RNA-seq results, 3 genes upregulated by *ARX* inhibition, encoding proteins involved in regulation of insulin release, were analyzed by qPCR (Fig. 5d). This analysis revealed changes in expression similar to those detected by the RNA-seq analysis.

### Effect of *ARX* inhibition in sorted expanded ACD cells

**Figure 6:**
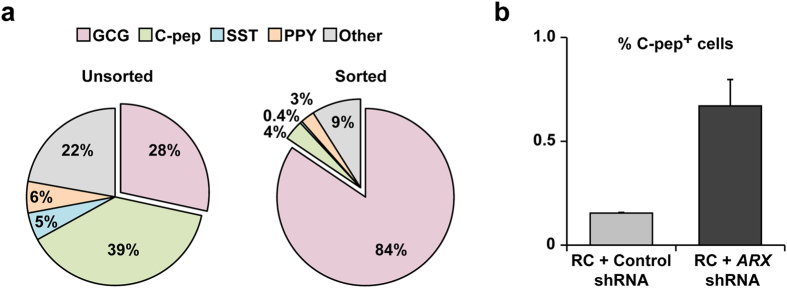
Effect of *ARX* inhibition in sorted expanded ACD cells. **(a)** Immunofluorescence analyses of islet endocrine cell types before and after sorting of α cells. Values are mean ± SE (n = 3 donors), based on counting >1000 cells per donor. (**b)** Immunofluorescence analyses of C-peptide in sorted α cells infected at passages 3–5 with *ARX* or control shRNA viruses, and treated with RC for 4 days. Values are mean ± SE (n = 3 donors), based on counting >1000 cells per donor.

α-cell-derived (ACD) cells present in the expanded islet cell population may be induced to redifferentiate into α cells following treatment with RC. To test the possibility of α-to-β-cell transdifferentiation as a result of *ARX* inhibition in these cells, α cells in dissociated uncultured islet cells were sorted to an average purity of 84.4% using an anti-α-cell antibody (Fig. 6a) and expanded in culture. Analysis of expanded ACD cells treated with *ARX* shRNA and RC showed an increase in the number of C-peptide^+^ cells (Fig. 6b); however, the percentage of C-peptide^+^ cells was very low. Considering that the sorted α-cell population contained 4% residual β cells, and that the percentage of BCD cells undergoing redifferentiation following *ARX* inhibition is 36.8% (Fig. 3b), redifferentiation of these cells alone would be expected to result in 1.3% C-peptide^+^ cells in the sorted cell population. Since the percentage of C-peptide^+^ cells was only 0.7% (Fig. 6b), we conclude that no ACD cells underwent transdifferentiation and contributed to the number of C-peptide^+^ cells.

## Discussion

Our findings demonstrate the expression of ARX in adult human islet cells, mostly but not exclusively in α cells. During islet cell expansion and dedifferentiation *in vitro ARX* is downregulated, and upregulated during expanded islet cell redifferentiation induced by RC. Using β-cell lineage tracing we show that ARX is upregulated in a fraction of BCD cells, supporting the possibility that BCD cells undergoing redifferentiation transition through a progenitor-like state, as also manifested by expression of transcription factors such as SOX9 and NGN3^7^. Upregulation of ARX likely interferes with BCD cell redifferentiation into β-like cells, since it inhibits expression of PAX4, a known activator of the *INS* gene.

*ARX* inhibition in BCD cells treated with RC potentiated their redifferentiation, as judged by a 12.8-fold increase in *INS* transcript levels and a significant upregulation of several other β-cell genes, compared with cells treated with RC alone, thus confirming the inhibitory effect of ARX. Silencing ARX also resulted in a 3.4-fold increase in *SST* transcript levels, which fits the notion of a common progenitor for β and δ cells. The 2.6-fold increase in the number of C-peptide^+^ cells likely represents those BCD cells which misexpressed ARX and regained their redifferentiation potential following inhibition of ARX. The percentage of ARX^+^ cells observed in immunostaining is probably lower than the actual percentage due to low sensitivity of the antibody. These results were reproducible in cells derived from multiple human donors.

Treatment of BCD cells with *ARX* shRNA and RC resulted in PAX4 upregulation at both mRNA and protein levels, while treatment of expanded islet cells with *PAX4* shRNA and RC resulted in upregulation of *ARX* transcript levels. These results demonstrate that the reciprocal repression between *Arx* and *Pax4* observed in mouse islet cells occurs in human cells as well.

Analyses of global transcriptome changes in BCD cells treated with *ARX* shRNA and RC revealed two main gene categories. In the first category *ARX* inhibition potentiated the effect (up- or downregulation) induced by RC alone, while in the second category *ARX* inhibition partly or completely counteracted the RC effect. Although none of the genes in either category is a known direct ARX target, further investigation of these genes may provide insights into the mechanisms mediating the effects of *ARX* inhibition on BCD cell phenotype, as well as the roles of ARX in islet cell development and function.

α cells have been suggested as a potential source for β-cell replacement, given their ability to transdifferentiate into β cells in a mouse model with a conditional loss-of-function mutation in the *Arx* gene^20^. In contrast, our findings in expanded human ACD cells treated with *ARX* shRNA and RC were unable to demonstrate α-cell transdifferentiation into β cells.

Our findings suggest that ARX inhibition may contribute to redifferentiation of expanded human BCD cells, as part of an approach for β-cell replacement therapy of diabetes based on *ex-vivo* expansion of islet cells from individual donors for transplantation into multiple recipients.

## Methods

### Ethics statement

This study was conducted according to the principles expressed in the Declaration of Helsinki. The Institutional Review Boards of the following medical centers, which provided human islets, each provided approval for the collection of samples and subsequent analysis: University of Geneva School of Medicine; San Raffaele Hospital, Milan; Faculty of Medicine, Lille 2 University; Massachusetts General Hospital; University of Pennsylvania; Scharp/Lacy Institute; University of Illinois; University of Wisconsin; University of Miami; University of Alberta; Southern California Islet Consortium. All donors provided written informed consent for the collection of all samples and subsequent analysis.

### Cell culture

Human islets were received 2–6 days following isolation. Islets from individual donors (see Supplementary Table S3) were dissociated into single cells and cultured in growth medium: CMRL 1066 medium containing 5.6 mM glucose and supplemented with 10% fetal bovine serum (FBS; HyClone, Logan, UT), 100 units/ml penicillin, 100 μg/ml streptomycin, 100 μg/ml gentamycin sulphate, and 5 μg/ml amphotericin B (all tissue culture reagents were from Biological Industries, Beit Haemek, Israel). The medium was replaced twice a week, and the cells were split 1:2 once a week. Lineage tracing was performed using the RIP-Cre/ER and pTrip–loxP-STOP-loxP-eGFP lentiviral vectors as described^5^. 4-hydroxytamoxifen (Sigma-Aldrich, St. Louis, MO) was added to a final concentration of 1 μM one day post-infection. Following overnight incubation the medium was changed to regular growth medium. Labeled cells were sorted using a FACS Aria sorter (Becton, Dickinson and Company, Franklin Lakes, NJ) as described^5^.

### *ARX* and *PAX4* inhibition by shRNA

*ARX* shRNAs (accession numbers TRCN00000163-33 and -34), *PAX4* shRNA (TRCN00000159-91) and a control empty shRNA (SHC001) cloned in pLKO.1-PURO lentiviral vector (Sigma-Aldrich) were used to produce virus particles as described^5^. Cells were infected at MOI 2:1 in CMRL 1066 medium containing 8 μg/ml polybrene (Sigma-Aldrich) overnight. The medium was then replaced with regular growth medium. Three days following infection the cells were selected with 1 μg/ml puromycin (Invivogen, Toulouse, France) for 3 days, and then used for analyses.

### Redifferentiation of expanded islet cells

Expanded human islet cells or sorted BCD cells were infected with *ARX* shRNA or control shRNA viruses. Six days following infection, cells were trypsinized, pelleted, and seeded at 4 × 10^4 ^cells/cm^2^ in ultra-low attachment plates (Corning, NY) in CMRL 1066 medium containing 5.6 mM glucose and supplemented with 100 U/ml penicillin, 100 μg/ml streptomycin, 1% BSA fraction V (Sigma-Aldrich), 1X insulin/transferrin/selenium (ITS; Gibco Life Technologies, Grand Island, NY), D-Glucose (final concentration 25 mM), 8 nM exendin-4 (Acris, Herford, Germany or Sigma-Aldrich), 8 nM activin A (PeproTech, Rocky Hill, NJ), 1X B27 supplement (Stem Cell Technologies, Vancouver, Canada or Gibco), and 10 mM nicotinamide (Sigma-Aldrich) (Redifferentiation Cocktail, RC) for 4–8 days. Half-volume medium changes were performed every other day.

### qPCR analysis

Total RNA was extracted using ZR RNA MiniPrep RNA Isolation Kit (Zymo Research, Irvine, CA), and treated with RNase-free DNase I (Thermo Scientific, Waltham, MA). cDNA was produced using High-Capacity cDNA Reverse Transcription Kit (Applied Biosystems, Foster City, CA). qPCR was carried out in triplicates using the Universal Probe Library System (Roche Diagnostics, Indianapolis, IN) in 7300 Real-time PCR system (Applied Biosystems). Results were normalized to TATA-box-binding protein (TBP) or Ribosomal protein large P0 (RPLP0) transcripts. These genes were selected as normalization controls since their detection threshold occurred at the same cycle in all the samples studied. Data analysis was performed with qbase+ software (Biogazelle, Zwijnaarde, Belgium). Supplementary Table S4 lists primer sequences. All reactions were performed with annealing at 60 °C. For undetectable transcripts, the cycle number was set to 40 for comparisons.

### Immunofluorescence analysis

Cells were spotted on slides using Shandon Cytospin4 centrifuge (Thermo Scientific), and fixed for 15 minutes at room temperature in 4% paraformaldehyde (Electron Microscopy Sciences, Hatfield, PA). Samples were blocked for 30 min at room temperature in blocking buffer [1% BSA (Sigma-Aldrich), 10% fetal goat serum (Biological Industries), and 0.2% saponin (Sigma-Aldrich)] and incubated overnight at 4 °C with primary antibodies (see Supplementary Table S5) diluted in blocking buffer. Slides were washed 5 times in PBS containing 0.1% Tween 20 (Sigma- Aldrich), and incubated for 40 min at room temperature with a secondary antibody conjugated to Alexa fluorophores (1:1000, Life Technologies). For rabbit anti-ARX, rabbit anti-PAX4, and mouse anti-PDX1 detection, samples were incubated with biotin-conjugated anti-rabbit/mouse IgG, respectively (1:200, Jackson ImmunoResearch, West Grove, PA) for 30 min at room temperature, followed by 40-min incubation at room temperature with Cy3-streptavidin (1:900, Jackson ImmunoResearch). The slides were mounted with Fluorescent Mounting Medium containing DAPI (GBI Labs, Bothell, WA). Images were taken using a Zeiss LTM 200 fluorescent microscope or a Leica SP5 confocal microscope. To demonstrate antibody specificity, a minus-primary antibody control was employed.

### Immunoblotting

Protein was extracted by incubating cells for 10 min in 50 mM Tris-HCl buffer, pH 7.4, containing 0.5% NP-40, 0.7% NaCl, 0.2% EDTA, and protease inhibitors (Roche Diagnostics). Samples of 20–40 μg protein were resolved by SDS-PAGE and transferred to Immobilon-P Membrane (Millipore, Billerica, MA). Non-specific sites were blocked for one hour at room temperature in blocking buffer containing 5% skim milk (Becton, Dickinson and Company) in TBST buffer. The membrane was then incubated with the antibodies listed in Supplementary Table S5. The bound antibody was visualized with the appropriate horseradish peroxidase-conjugated anti-IgG (Jackson ImmunoResearch) and SuperSignal West Chemiluminescent Substrate kit (Thermo Scientific). Signal intensity was quantitated using TINA 2.0 software.

### C-peptide assay

Cells were lysed in acidic alcohol, and the supernatant was taken for analysis of C-peptide content. Human C-peptide was quantified using an ultrasensitive ELISA kit (Mercodia, Uppsala, Sweden; sensitivity 1.5 pmol/L; crossreactivity with insulin and proinsulin 0.0006% and 1.8%, respectively) according to the manufacturer’s protocol.

### Transcriptome analyses

Total RNA was extracted using the ZR RNA MiniPrep RNA Isolation Kit (Zymo Research), and treated with RNase-free DNase I (Thermo Scientific). cDNA libraries were constructed following TruSeq Stranded mRNA Library Prep Kit (Illumina, San Diego, CA) for next-generation sequencing. Briefly, 400 ng total RNA was used as starting material and PolyA-selected mRNAs were purified, size-fractioned, and subsequently transcribed into single-stranded cDNA by random hexamer priming. Following second-strand synthesis, double-stranded cDNAs were blunt-end fragmented and indexed using adapter ligation, after which they were amplified and sequenced according to manufacturer’s protocol (Illumina). Libraries were pooled and sequenced on one 50-bp single-read HiSeq 2500 Ultra-High-Throughput Sequencing System (Illumina) lane with MiSeq Reagent Kit v3 (Illumina). Standard quality checks for material degradation (Bioanalyzer, Agilent Technologies, Santa Clara, CA) and concentration (Qubit, Life Technologies) were done before and after library construction, ensuring that samples were suitable for sequencing. RNA-seq reads were mapped to the human genome using TopHat2^29^. All samples had more than 20M uniquely mapped reads. Gene expression levels were calculated using HTseq-count utility^30^ using Genecode gene annotations (v19)^31^. Only genes that were readily detected in the dataset (covered by at least 20 reads; 14,647 genes) were included in subsequent analyses. Quantile normalization was used to normalize expression levels across samples^32^. False discovery rate (FDR) was estimated by 1,000 random gene permutations of the data. Cluster analysis was performed using the CLICK algorithm^33^ implemented in the EXPANDER package for gene expression analysis^34^. Heatmaps were created using the gplots R package. Functional enrichment analysis was carried out using DAVID^35^. The set of all genes expressed in the dataset was used as the background set in this analysis. The data was deposited in the GEO database (accession number GSE73433).

### Alpha Cell Sorting

Dissociated islet cells were blocked for 20 min at 4 °C in blocking buffer (10% FBS in PBS) and incubated for 30 min at 4 °C with mouse-IgM anti-human-a-cells (Beta Cell Biology Consortium, Nashville, TN). Cells were washed 3 times in MACS wash buffer (Miltenyi Biotec, Bergisch Gladbach, Germany), incubated for 15 min at 4 °C with anti-mouse IgM MicroBeads (Miltenyi Biotec), washed, and loaded onto a MACS MS column (Miltenyi Biotec). The positive fraction was then cultured as described above.

### Statistical Analysis

Significance was determined using Student’s t test. To approach a normal distribution of the qPCR data, a logarithmic transformation was performed.

## Additional Information

**How to cite this article**: Friedman-Mazursky, O. *et al*. Redifferentiation of expanded human islet β cells by inhibition of ARX. *Sci. Rep*. **6**, 20698; doi: 10.1038/srep20698 (2016).

## Supplementary Material

Supplementary Information
