# Supplementary material for: Redifferentiation of expanded human islet β cells by inhibition of ARX
Source: Sci Rep. 2016 Feb 9;6:20698. doi: 10.1038/srep20698 (PMC4746595; doi:10.1038/srep20698)
Supplement: Supplementary Information [file srep20698-s1.doc]

Redifferentiation of expanded human islet β cells by inhibition of ARX

Orr Friedman-Mazursky, Ran Elkon and Shimon Efrat

**Supplementary Table S1. Genes upregulated >1.5-fold in cells treated with RC and *ARX* shRNA, compared with RC+control shRNA.** The list is based on the RNA-seq analyses shown in Fig. 5a

**Supplementary Table S2. Genes downregulated >1.5-fold in cells treated with RC and *ARX* shRNA, compared with RC+control shRNA.** The list is based on the RNA-seq analyses shown in Fig. 5b.

**Supplementary Table S3.** Islet donors used in this study

| **Donor No.** | **Donor Sex** | **Donor Age (y)** | **Donor BMI** | **Islet Purity (%)** |
| --- | --- | --- | --- | --- |
| **1** | m | 50 | 26.0 | 90 |
| **2** | m | 21 | 33.8 | 85 |
| **3** | f | 32 | 26.9 | 80 |
| **4** | f | 44 | 21.5 | 80 |
| **5** | m | 38 | 29.8 | 85 |
| **6** | f | 55 | 25.4 | 85 |
| **7** | f | 49 | 27.1 | 90 |
| **8** | m | 56 | 30.8 | 90 |
| **9** | m | 62 | 26.0 | 90 |
| **10** | f | 48 | 22.0 | 95 |
| **11** | m | 65 | 23.5 | 85 |
| **12** | m | 46 | 24.0 | 74 |
| **13** | m | 49 | 31.1 | 90 |
| **14** | f | 20 | 24.6 | 85 |
| **15** | f | 51 | 21.2 | 85 |
| **16** | m | 55 | 33.6 | 92 |
| **17** | m | 63 | 24.5 | 85 |
| **18** | m | 55 | 27.0 | 80 |
| **19** | m | 43 | 34.7 | 80 |
| **20** | f | 47 | 20.6 | 90 |
| **21** | m | 31 | 29.0 | 85 |
| **22** | m | 14 | 27.1 | 80 |
| **23** | m | 27 | 20.2 | 85 |
| **24** | m | 51 | 26.8 | 95 |
| **25** | m | 32 | 25.7 | 70 |
| **26** | m | 42 | 22.8 | 95 |
| **27** | f | 32 | 27.4 | 80 |
| **28** | f | 60 | 34.5 | 80 |
| **29** | f | 51 | 28.7 | 88 |
| **30** | m | 62 | 28.0 | 80 |
| **31** | f | 61 | 31.1 | 90 |
| **32** | f | 29 | 21.0 | 90 |
| **33** | f | 48 | 32.8 | 90 |
| **34** | f | 52 | 31.4 | 80 |
| **35** | m | 48 | 30.7 | 80 |
| **36** | m | 15 | 23.0 | 90 |
| **37** | m | 26 | 26.8 | 70 |
| **38** | m | 55 | 33.5 | 85 |
| **39** | f | 60 | 22.0 | 90 |
| **40** | m | 53 | 20.1 | 95 |
| **41** | m | 22 | 32.9 | 90 |
| **42** | m | 19 | 34.1 | 95 |
| **43** | f | 54 | 24.2 | 38 |
| **Mean ± SD** |  | **44 ± 15** | **27 ± 4** | **85 ± 10** |

**Supplementary Table S4.** Primer sequences for qPCR analyses

| **Gene Symbol** | **Sense Primer** | **Antisense Primer** |
| --- | --- | --- |
| ***ABCC8*** | gcactttccgcatcttgg | aaaccccgagaaattgtgtct |
| ***ARX*** | gcaccacgttcaccagcta | cagcctcatggccagttc |
| ***GHRL*** | ggggaagtttcttcaggaca | cttgtgggcgatcacttgt |
| ***GCG*** | gtacaaggcagctggcaac | tgggaagctgagaatgatctg |
| ***IAPP*** | ttaccaaattgtagaggctttcg | ccctgcctctatacactcactacc |
| ***INS*** | aggcttcttctacacacccaag | cacaatgccacgcttctg |
| ***MAFA*** | agcgagaagtgccaactcc | ttgtacaggtcccgctcttt |
| ***MAFB*** | agggaagctgccaagctc | atttgaccataagacaaggctgt |
| ***NEUROD1*** | ctgctcaggacctactaacaacaa | gtccagcttggaggacctt |
| ***NGN3*** | ttttgcgccggtagaaag | gggcaggtcacttcgtctt |
| ***NKX2.2*** | cgagggccttcagtactcc | ggggacttggagcttgagt |
| ***NKX6.1*** | cgttggggatgacagagagt | cgagtcctgcttcttcttgg |
| ***PPY*** | tctagtgcccatttactctggac | gcaggtggacaggagcag |
| ***PAX4*** | caggaggaccagggactacc | gagccactatggggagtgag |
| ***PAX6*** | ctttcatttgttctgtgcaacat | ccccaggctttttagtgaagt |
| ***PDX1*** | cacatccctgccctcctac | gaagagccggcttctctaaac |
| ***RAB15*** | tagatggtggaaagcaaagga | tccttttctcattagccagtgatt |
| ***RPLPO*** | tctacaaccctgaagtgcttgat | caatctgcagacagacactgg |
| ***SST*** | accccagactccgtcagttt | acagcagctctgccaagaag |
| ***SULF2*** | gtgacagcggggactacaag | cgactgcggacatagctg |
| ***SYT13*** | tccttgatggagtcccaaaa | accctgatggttgaggtgtc |
| ***TBP*** | cggctgtttaacttcgcttc | cacacgccaagaaacagtga |

**Supplementary Table S5.** Primary antibodies for immunofluorescence (IF), immunoblotting (IB) and magnetic activated cell sorting (MACS) analyses

| **Antigen** | **Species** | **Manufacturer** | **Working Dilution** |
| --- | --- | --- | --- |
| **α cells** | mouse | Beta Cell Biology Consortium | 1:1 MACS |
| **ARX** | rabbit | A gift from Dr. Kunio Kitamura^33^ | 1:500 IF |
| **ARX** | rabbit | A gift from Dr. Patrick Collombat^15^ | 1:1000 IB |
| **C-peptide (human)** | rat | Beta Cell Biology Consortium | 1:1000 IF |
| **C-peptide (human)** | mouse | Sigma-Aldrich | 1:500 IF |
| **HSC70** | mouse | Santa Cruz Biotechnology | 1:1000 IB |
| **GCG** | mouse | Sigma-Aldrich | 1:1000 IF |
| **PAX4** | rabbit | A gift from Dr. Beatriz Sosa-Pineda^14^ | 1:500 IF  1:2000 IB |
| **PDX1** | mouse | R & D Systems | 1:500 IF |
| **PPY** | rabbit | Zymed | 1:100 IF |
| **SST** | rabbit | Dako | 1:200 IF |


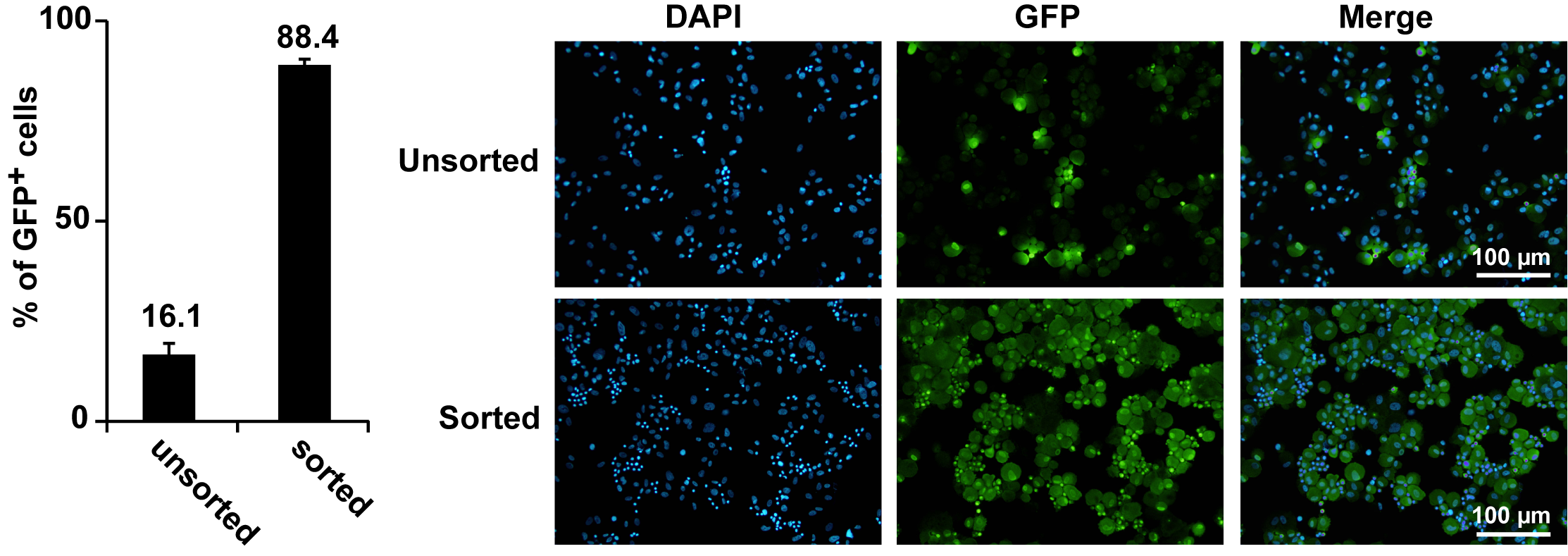
**Supplementary Fig. S1. Sorting of GFP^+^ cells.** Immunofluorescence analysis of GFP in expanded islet cells at passage 3 before and after cell sorting. Data are mean±SE (n=5 donors).


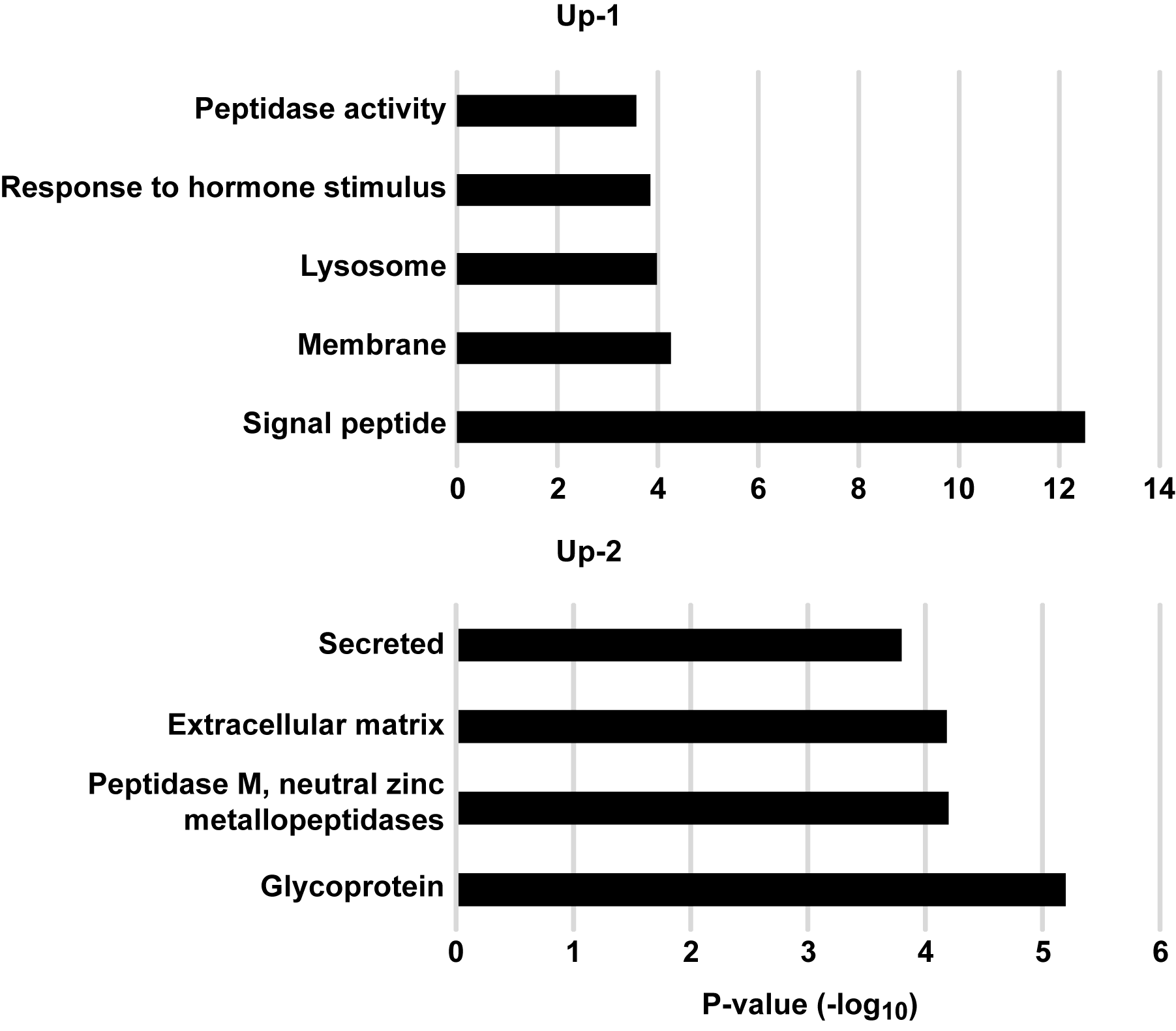


**Supplementary Fig. S2.** **Enriched functional categories in gene clusters in Fig. 5a.**
